# The potential mechanism of huazhuojiedu decoction in the treatment of ulcerative colitis based on network pharmacology and experimental validation

**DOI:** 10.3389/fphar.2022.1033874

**Published:** 2022-10-14

**Authors:** Xuemei Jia, Ze Li, Yuxi Guo, Hongyu Ma, Jie Wang, Yucong Xue, Bolin Li, Yanru Cai, Qian Yang

**Affiliations:** ^1^ The First Affiliated Hospital, Hebei University of Chinese Medicine, Shijiazhuang, China; ^2^ Department of Gastroenterology, Hebei Province Hospital of Chinese Medicine, Shijiazhuang, China; ^3^ Department of Traditional Chinese Medicine, Hebei General Hospital, Shijiazhuang, China; ^4^ School of Pharmacy, Hebei University of Chinese Medicine, Shijiazhuang, China

**Keywords:** huazhuojiedu decoction, ulcerative colitis, network pharmacology, inflammation, oxidative stress, NLRP3/caspase-1 signaling pathway, pyroptosis

## Abstract

Huazhuojiedu decoction (HZJDD), a traditional Chinese medicine prescription, has been clinically proven to be an effective treatment for ulcerative colitis (UC). However, the mechanism of HZJDD in the treatment of UC remains unclear. This study combined network pharmacology with experimental validation to explore the potential mechanism of HZJDD on UC. First, the relationship network diagrams between HZJDD and UC were established based on multiple databases. Then, the HZJDD-UC intersection genes target network was constructed and Gene Ontology-Biological processes (GO-BP) analysis was performed to discover the potential pharmacological mechanism. Finally, the results of GO-BP were verified in dextran sulfate sodium salt (DSS) induced UC rats. The network pharmacology results showed that 119 active components and 146 potential targets were screened for HZJDD, and six of the top 15 biological processes belonged to inflammatory response, cellular response to hypoxia, and cellular response to lipopolysaccharide (LPS). The GO-BP results indicated that the mechanism of HZJDD treatment of UC was related to inflammation, oxidative stress, and the regulation of LPS. Animal experiments showed that HZJDD could significantly reduce the disease activity index (DAI) score, improve colon length, and effectively repair the histomorphological and micromorphological changes in DSS-induced UC rats. Moreover, HZJDD reduced the expressions of CRP, TNF-α, IL-6, LPS, IL-1β, and IL-18; downregulated the activity of MDA; and upregulated the activities of CAT, GSH, and SOD in DSS-induced UC rats. Furthermore, HZJDD suppressed the expression of the NLRP3/caspase-1 signaling pathway at the gene and protein levels to inhibit pyroptosis. Network pharmacology and animal experiments showed that HZJDD exerted a therapeutic effect on DSS-induced UC rats by reducing inflammation, oxidative stress, and restraining the NLRP3/caspase-1 signaling pathway to inhibit pyroptosis.

## 1 Introduction

Ulcerative colitis (UC) is a recurrent chronic inflammatory bowel disease characterized by mucosal inflammatory infiltration and intestinal wall damage (Ungaro et al., 2017). Epidemiology shows that UC has become a global disease (Liu et al., 2022). In addition, UC patients are at increased risk of developing colorectal cancer (Wang B. et al., 2021). It is currently believed that UC is caused by a complex interaction of genetic susceptibility and stimulation of environmental triggers leading to dysregulation of the immune system; although researchers continue to focus on environmental exposures, genes, gut microbiota, and the pathogenicity of UC, the exact mechanism has not been clearly explained (Du and Ha, 2020).

Drug therapy is the preferred treatment for UC, including 5-aminosalicylic acid (5-ASA), corticosteroids, and immunosuppressants. However, these drug treatments have many limitations, such as unavoidable adverse reactions and a heavy economic burden (Rosenberg and Peppercorn, 2010). Consequently, exploring a series of safe, efficient, and compliant treatment methods is crucial. Several previous studies have found that the massive release of pro-inflammatory mediators (CRP, TNF-α, IL-6, LPS) and the disturbance of colonic antioxidant capacity can induce the initiation of UC (Owusu et al., 2020; Ansari et al., 2021). Inflammation and oxidative stress-induced cell death, including pyroptosis (Xie et al., 2020). Notably, inflammatory response and oxidative damage are important links in NLRP3-mediated pyroptosis (Chi et al., 2022). There are increasing studies on pyroptosis in gastrointestinal diseases, and researchers are gradually realizing the particular connection between pyroptosis and UC (Zhang et al., 2020). The NLRP3/caspase-1 signaling pathway is a classical signaling pathway for pyroptosis (Burdette et al., 2021). Shigui Xue et al. found that inhibition of the NLRP3/caspase-1 pathway was an effective way to improve DSS rats (Xue et al., 2022), which was supported by data from Hu et al. (2021). The NLRP3/caspase-1 pathway exerts a vital role in UC by regulating pyroptosis.

Traditional Chinese medicine (TCM) has been proven to be one of the most promising ways to treat UC with the advantages of reliable curative effect and few adverse reactions (Liu et al., 2022; Zhang et al., 2022). According to TCM theory, UC is attributed to “diarrhea” and “dysentery” because of its diarrhea and abdominal pain symptoms, which are caused by damp heat and dietary damage (Wang et al., 2021d). Notably, Huazhuojiedu decoction (HZJDD) was formed by the evolution of the classical Chinese formulas Baitouweng decoction (Miao et al., 2020; Xuan-Qing et al., 2021), Shaoyao decoction (Wei et al., 2021) and Xianglian pill (Liu C. S. et al., 2021). Based on the theory of TCM, HZJDD has the effects of “clearing heat”, “eliminating turbid fluid” and “removing toxicity”. Our clinical studies showed that HZJDD can effectively relieve the symptoms of abdominal pain and diarrhea in UC patients, reduce inflammatory reaction, and play a key role in the treatment of mild to moderate UC. HZJDD is composed of 17 kinds of medicinal herbs, including *Coptidis Rhizoma*, *Amomi Fructus*, *Herba Patriniae*, *Fraxini Cortex*, *Sanguisorbae Radix*, *Pteridis*, *Multifidae Herba*, *Pulsatiliae Radix*, *Bupleuri Radix*, *Angelicae Sinensis Radix*, *Paeoniae Radix Alba*, *Aucklandiae Radix*, *Atractylodis Macrocephalae Rhizoma*, *Euryales Semen*, *Catechu*, *Coicis semen*, *Schisandrae Chinensis Fructus*, *Cuscutae Semen*. As a multi-components and multi-target drug, HZJDD achieves a therapeutic effect by modulating a molecular network with active components. However, the potential target and comprehensive mechanism of HZJDD in treating UC remain unclear.

Network pharmacology is a new method of systematic network analysis that can observe the interrelationships among active drug ingredients, proteins, genes and diseases at the molecular level (Wang et al., 2022). The advantages of its integrity and systematic coincide with the characteristics of multi-component and multi-target TCM compounds, and it has been proven to be a powerful approach to TCM research (Zhang et al., 2019; Wang X. et al., 2021). In addition, the application of network pharmacology is a new paradigm of TCM from empirical medicine to evidence-based medicine (Yang et al., 2022).

Based on the above, this study used network pharmacology to predict the potential biological process of HZJDD in the treatment of UC and conducted animal experiments to evaluate the effects of HZJDD on inflammation, oxidative stress, and the NLRP3/caspase-1 signaling pathway.

## 2 Materials and methods

### 2.1 Network pharmacology analysis

#### 2.1.1 Collection of active components and prediction of potential targets of HZJDD

The chemical components information of HZJDD was searched through the Traditional Chinese Medicine System Pharmacology Database and Analysis Platform (TCMSP, https://old.tcmsp-e.com/tcmsp.php). Oral bioavailability (OB) ≥ 30% and drug-likeness (DL) ≥ 0.18 were the initial screening criteria. The obtained drug targets were standardized by the UniProtKB database, and the drug targets were converted into corresponding gene names. Then, using “Ulcerative Colitis” as the keyword, UC-related target information was retrieved from Genecards and OMIM databases and normalized using the UniProtKB database. Finally, the intersection and intersection target plot of HZJDD and UC targets were gained by the venny analysis tool (https://bioinfogp.cnb.csic.es/tools/venny/).

#### 2.1.2 Biological processes analysis of HZJDD-UC intersection targets

The intersection targets of HZJDD-UC were imported into the DAVID 6.8 (https://david.ncifcrf.gov/) database for Gene Ontology-Biological processes (GO-BP) enrichment analysis. The gene name was selected as “OFFICE_GENE_SYMBOL”, the species was selected as “*Homo sapiens*”, and the GO database was selected as “GOTERM_BP_DIRECT”. *p* < 0.05 was used as the screening criterion. We sorted the *p*-values from small to large, selected the first 15 analysis results, and uploaded them to the WeChat cloud platform (http://www.bioinformatics.com.cn/?p=1) for data visualization, with a the circle as a symbol for the number of genes and the color as a symbol for enrichment significance.

### 2.2 Preparation of the HZJDD

HZJDD formula granules were supplied by E-FONG Pharmaceutical Co., Ltd. (Guangdong, China), comprised 17 commonly used herbs: *Coptidis Rhizoma* (no.1100053), *Amomi Fructus* (no.1095333), *Herba Patriniae* (no.1055843), *Fraxini Cortex* (no.1045433), *Sanguisorbae Radix* (no.1096053), *Pteridis Multifidae Herba* (no.0116503), *Pulsatiliae Radix* (no.1098083), *Bupleuri Radix* (no.1093003), *Angelicae Sinensis Radix* (no.1108183), *Paeoniae Radix Alba* (no.1100023), *Aucklandiae Radix* (no.1043103), *Atractylodis Macrocephalae Rhizoma* (no.1071003), *Euryales Semen* (no.1090073), *Catechu* (no.1096763), *Coicis semen* (no.1102383), *Schisandrae Chinensis Fructus* (no.1081463), *Cuscutae Semen* (no.1051353). All formulation granules of HZJDD were mixed according to the dosage in Table 1 and dissolved in distilled water at 100°C to prepare suspensions with concentrations of 4, 2 and 1 g/ml.

**TABLE 1:** The composition and clinical dosage of HZJDD.

English name	Mandarin name	g/day
Coptidis Rhizoma	Huanglian	6
Amomi Fructus	Sharen	6
Herba Patriniae	Bai jiangcao	15
Fraxini Cortex	Qianpi	12
Sanguisorbae Radix	Diyu	15
Pteridis Multifidae Herba	Fengweicao	15
Pulsatiliae Radix	Baitouweng	12
Bupleuri Radix	Chaihu	6
Angelicae Sinensis Radix	Danggui	10
Paeoniae Radix Alba	Baishao	20
Aucklandiae Radix	Muxiang	6
Atractylodis Macrocephalae Rhizoma	Baizhu	12
Euryales Semen	Qianshi	15
Catechu	Ercha	6
Coicis semen	Yiyiren	20
Schisandrae Chinensis Fructus	Wuweizi	6
Cuscutae Semen	Tusizi	10

#### 2.2.1 Drugs and chemical reagents

Mesalazine (Dr.Falk Pharma GmbH, H2017158) was purchased from Hebei Hospital of Traditional Chinese Medicine (Hebei, China). Mesalazine was dissolved in distilled water to prepare a suspension with a concentration of 0.05 g/ml (Li B. et al., 2021). Dextran sulfate sodium salt (DSS) was purchased from Yeasen Biotechnology Co., Ltd. (Shanghai, China). DSS suspension (3.5%) was prepared by dissolving 3.5 g of DSS in 100 ml of distilled water.

### 2.3 Animal experiment

#### 2.3.1 Experimentals animals and modeling

Sixty SPF grade male Wistar rats (160 ± 20 g, 6–8 weeks old) were provided by SiPeiFuBiotechnology Co., Ltd. [Beijing, China; Certificate NO. SCXK (Jing) 2019-0010], and entered the experiment after 7 days of adaptive feeding.

The sixty rats were divided (random number table method) into a normal group (NG) with ten rats and a treatment group with 50 rats. According to our previous experiments (Li F. et al., 2021; Jia et al., 2022), rats in NG were routinely fed, and rats in the treatment group were given a 3.5% DSS suspension solution (a fresh solution was prepared every day) for ten consecutive days to obtain UC. Fifty successfully modeled rats were randomly divided into five groups (n = 10): model group (MG), western medicine group (WG), high-dose HZJDD (H-HG, 40 g/kg), middle-dose HZJDD (M-HG, 20 g/kg), and low-dose HZJDD (L-HG, 10 g/kg). The adult dose of HZJDD was 192 g/d. By calculating the dose conversion coefficient per kilogram of body weight between animals and humans (Phok et al., 2017; Du et al., 2021), when the adult standard body weight is 60 kg, the equivalent dose of HZJDD in rats was about 20 g/kg/d, so the doses in this study were 10, 20, and 40 g/kg/d respectively (Yan et al., 2020).

NG and MG were given normal saline by gavage, WG was given mesalazine suspension (0.315 g/kg) by gavage (Jia et al., 2022), and each HZJDD treatment group was given a corresponding dose of HZJDD suspension by gavage, once per day for each group.

After 2 weeks of treatment, the rats were fasted for 24 h and were fixed on the operating table with 1% pentobarbital (50 mg/kg) after intraperitoneal injection (Zhao et al., 2022). Five milliliters of abdominal aortic blood were collected and placed into the test tube, centrifuged at 3,000 r/min at 4°C for 15 min, and the separated supernatant was placed into the refrigerator at −80°C for testing. The carcasses of experimental rats were treated without pollution. The Ethics Committee of Hebei University of Traditional Chinese Medicine approved this experiment (DWLL202208002).

#### 2.3.2 Histological examination and transmission electron microscopy

The colon tissues with obvious lesions were fixed in 4% paraformaldehyde solution, dehydrated, embedded in paraffin, and sectioned. Then, tissues were dewaxed, rehydrated, and stained. The plates were sealed with neutral resin, and the results were observed under light microscopy.

Fresh colon tissues were cut and fixed in 4% glutaraldehyde, fixed with osmic acid, dehydrated with acetone gradient, embedded, and then ultrathin sectioned. Then, sections were double-stained with saturated a aqueous solution of uranyl acetate and lead citrate, observed by transmission electron microscope, and images collected for analysis.

#### 2.3.3 Disease activity index score

Colon lesions in each group were assessed using the DAI score. DAI calculation formula (Lv et al., 2021) (weight loss score + stool trait score + stool blood score)/3 (Supplementary Table S1).

#### 2.3.4 Enzyme-linked immunosorbent assay

According to the product instructions, the levels of CRP (EK394-96), and LPS (CSB-E14247r) in serum and TNF-α (EK382/3-96), IL-6 (EK306/3-96), IL-1β (EK301B/3-96), and IL-18 (CSB-E04610r) in colon tissues of rats were detected by ELISA. CRP, IL-6, TNF-α, and IL-1β ELISA kits were supplied by Multiscences (Lianke) Biotech Co., Ltd. (Zhejiang, China). LPS and IL-18 kits were supplied by Cusabio Biotech Co., Ltd. (Wuhan, China).

#### 2.3.5 Biochemical analysis

According to the kit instructions, the activity of SOD (A001-1), CAT (A007-1), GSH (A006-2) and MDA (A003-1) in rats’ colon tissues were determined. All kits were supplied by Nanjing Jiancheng Bioengineering Institute (Nanjing, China).

#### 2.3.6 Western blot analysis

Total protein was collected from rats’ colon tissues after homogenization, lysis and centrifugation. Protein concentration was measured with a BCA protein concentration assay kit (Servicebio Technology Co., Ltd., Wuhan, China). An equal amount of protein was analyzed by SDS-PAGE gels (Servicebio Technology Co., Ltd., Wuhan, China), and the protein on the gel was transferred to the PVDF membrane; skim milk was added to the block for 30 min on a destaining shaker. Primary antibodies were diluted proportionally and incubated overnight at 4°C: NLRP3 (1:1,000, BA3677, Boster Biological Technology Co., Ltd., California, United States), ASC (1:1,000, BS-6741R, BIOSS Inc., Beijing, China), caspase-1 (1:1,000, GB11383, Servicebio Technology Co., Ltd., Wuhan, China), cleaved-caspase-1 (1:1,000, 4,199, Cell Signaling Technology, Beverly, MA, United States), GSDMD (1:1,000, AB219800, Abcam, Cambridge, United Kingdom), GSDMD-N (1:1,000, Ab215203, Abcam, Cambridge, United Kingdom), and β-actin (1:2000, GB15001, Servicebio Technology Co., Ltd., Wuhan, China). After the secondary antibody (1:5,000, GB23303, Servicebio Technology Co., Ltd., Wuhan, China) was diluted in proportion, shaken slowly on a shaker and incubated at room temperature for 30 min. The optical density values of the target band and β-actin were measured by ImageJ analysis software. All proteins were compared and normalized to β-actin.

#### 2.3.7 RNA extraction and quantitative real-time PCR

An appropriate amount of tissue was added to the Trizol reagent and placed on ice to fully lyse, and total RNA was extracted. The concentration and purity of total RNA were detected by Nanodrop 2000, and the PCR reaction system was prepared for PCR amplification. Gene expression analysis was performed using the 2^-△△CT^ quantitative method. Primer sequences are detailed in Supplementary Table S2.

### 2.4 Statistical analysis

Data were expressed as mean ± standard deviation (SD) and analyzed by SPSS 23.0 software. Data conforming to normal distribution and homogeneity of variance were analyzed by one-way ANOVA followed by LSD’s multiple comparison test. *p*-values < 0.05 were considered significant.

## 3 Results

### 3.1 Network pharmacology analysis of HZJDD in the treatment of UC

#### 3.1.1 Active components and potential targets of HZJDD

Using the TCMSP database, we searched the chemical components of HZJDD, screened with OB ≥ 30% and DL ≥ 0.18, and eliminated the chemical components that had no effect, and finally got 119 chemical components. Among them, 11 components were in *Coptidis Rhizoma*, eight components were in *Amomi Fructus*, 11 components were in *Herba Patriniae*, three components were in *Fraxini Cortex*, nine components were in *Sanguisorbae Radix*, two components were in *Pteridis Multifidae Herba*, nine components were in *Pulsatiliae Radix*, 12 components were in *Bupleuri Radix*, two components were in *Angelicae Sinensis Radix*, eight components were in *Paeoniae Radix Alba*, five components were in *Aucklandiae Radix*, four components were in *Atractylodis Macrocephalae Rhizoma*, one component was in *Euryales Semen*, 11 components were in *Catechu*, six components were in *Coicis semen*, eight components were in *Schisandrae Chinensis Fructus*, and ten components were in *Cuscutae Semen* (Supplementary Table S3). The network topology analysis was performed on 119 active compounds with degree as the filtering condition. Degree is the most direct and important parameter to measure the nodes (Zhang et al., 2015). The larger the degree of a node means the more important the node is in the network. We found that six of the active ingredients showed a preeminent position, including quercetin, kaempferol, beta-sitosterol, stigmasterol, luteolin, isorhamnetin (Supplementary Table S7). Two-hundred-twelve single targets were obtained after deduplicating HZJDD (Supplementary Table S4).

#### 3.1.2 Targets related to UC

**FIGURE 1:**
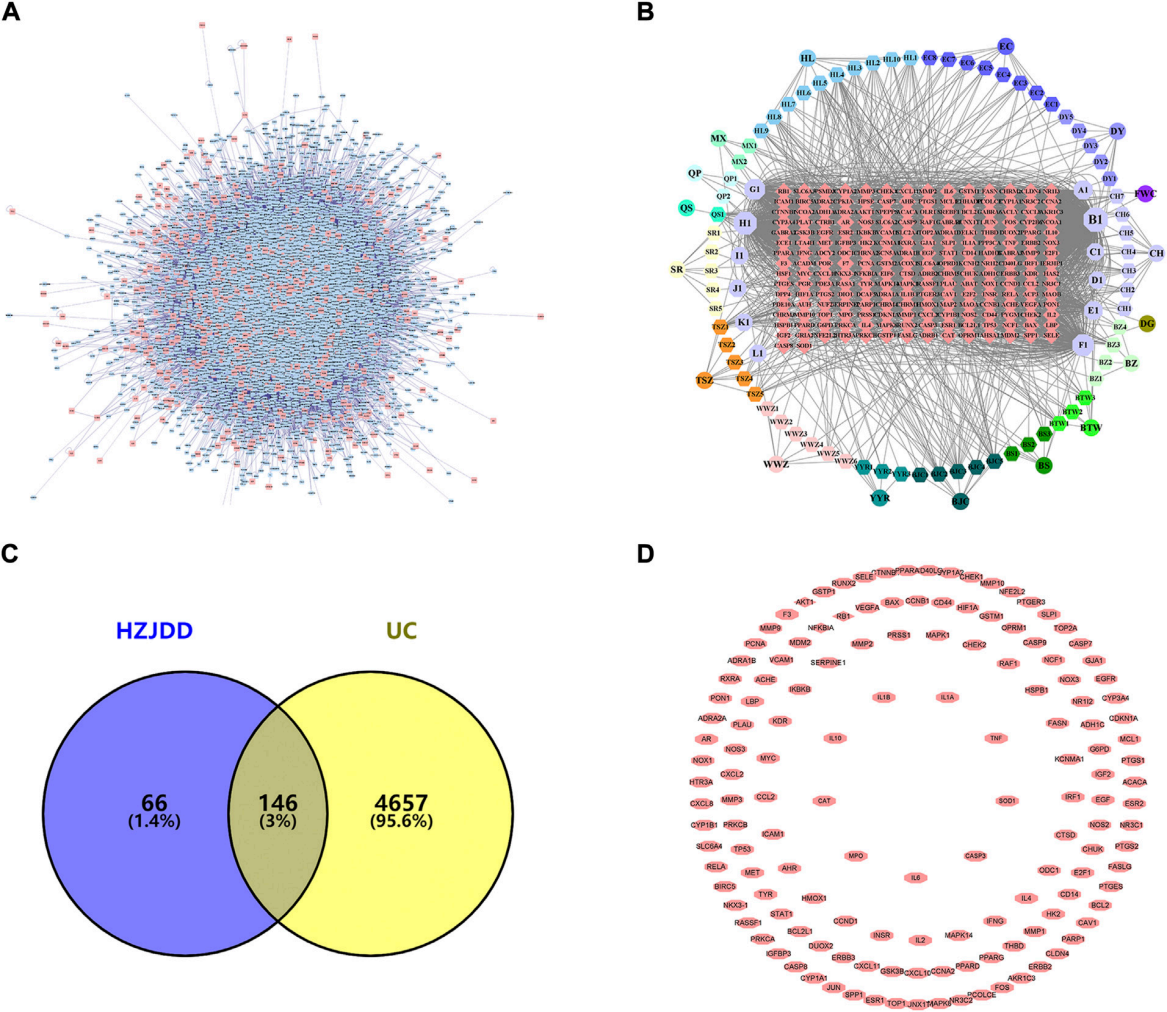
PPI network of HZJDD-UC. **(A)** The PPI network of UC targets. **(B)** The HZJDD-target network. **(C)** The common targets of HZJDD-UC. **(D)** The PPI network of HZJDD-UC common targets.

The Genecards and OMIM databases were searched with “Ulcerative Colitis” as the keyword, and 4803 UC-related targets (Figure 1A) were obtained by removing duplicate targets (Supplementary Table S5). The intersection of HZJDD and UC targets resulted in 146 intersection targets (Figure 1C) (Supplementary Table S6).

The “HZJDD” (Figure 1B) and “HZJDD-UC” (Figure 1D) intersection target network diagrams were constructed and analyzed using Cytoscape 3.8.2. The results showed that HZJDD has abundant therapeutic targets, which fully reflected that HZJDD interferes with UC through multi-target and multi-center methods.

#### 3.1.3 Go analysis and construction of PPI network

**FIGURE 2:**
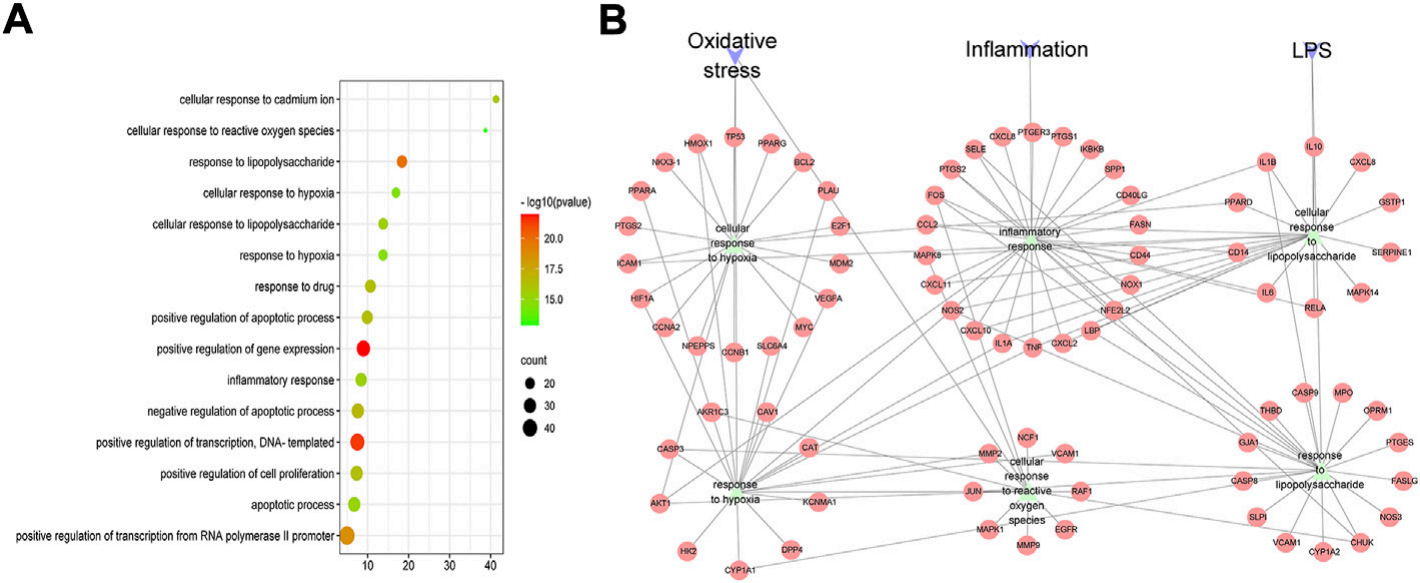
GO-BP analysis of HZJDD-UC common targets. **(A)** Bubble chart of the first 15 biological processes. **(B)** PPI network of “inflammation, oxidative stress, and lipopolysaccharide” biological process and HZJDD-UC common targets.

The biological processes of 146 therapeutic targets were analyzed by the DAVID database. The results showed that potential therapeutic targets were mainly enriched in biological processes such as positive regulation of transcription, response to lipopolysaccharide (LPS), and response to hypoxia. We analyzed the first 15 biological processes and found that six of them belonged to the categories of the inflammatory response, cellular response to hypoxia and response to LPS, suggesting that the anti-UC mechanism of HZJDD may be related to inflammation, oxidative stress and the regulation of LPS (Figure 2A).

The PPI network of “inflammation, oxidative stress and LPS” biological processes and intersection targets were constructed, and the network topology was analyzed. The key targets were screened by degree, which is the most direct and important parameter to measure the nodes. NOS2, IL-1β, TNF-α, IL-6, and LPS were the key targets with high degree-ranking (Supplementary Table S8). We found that TNF-α and IL-6 are related to inflammation; SOD and CAT are related to oxidative stress; and IL-6 and IL-1β are associated with LPS-induced cellular responses (Figure 2B). Therefore, combined with the results of PPI (Figure 2B), we conducted animal experiments to detect the biological processes of inflammation, oxidative stress, and LPS-induced cellular responses.

### 3.2 Results of animal experiments

#### 3.2.1 HZJDD alleviated DSS-induced UC in rats

**FIGURE 3:**
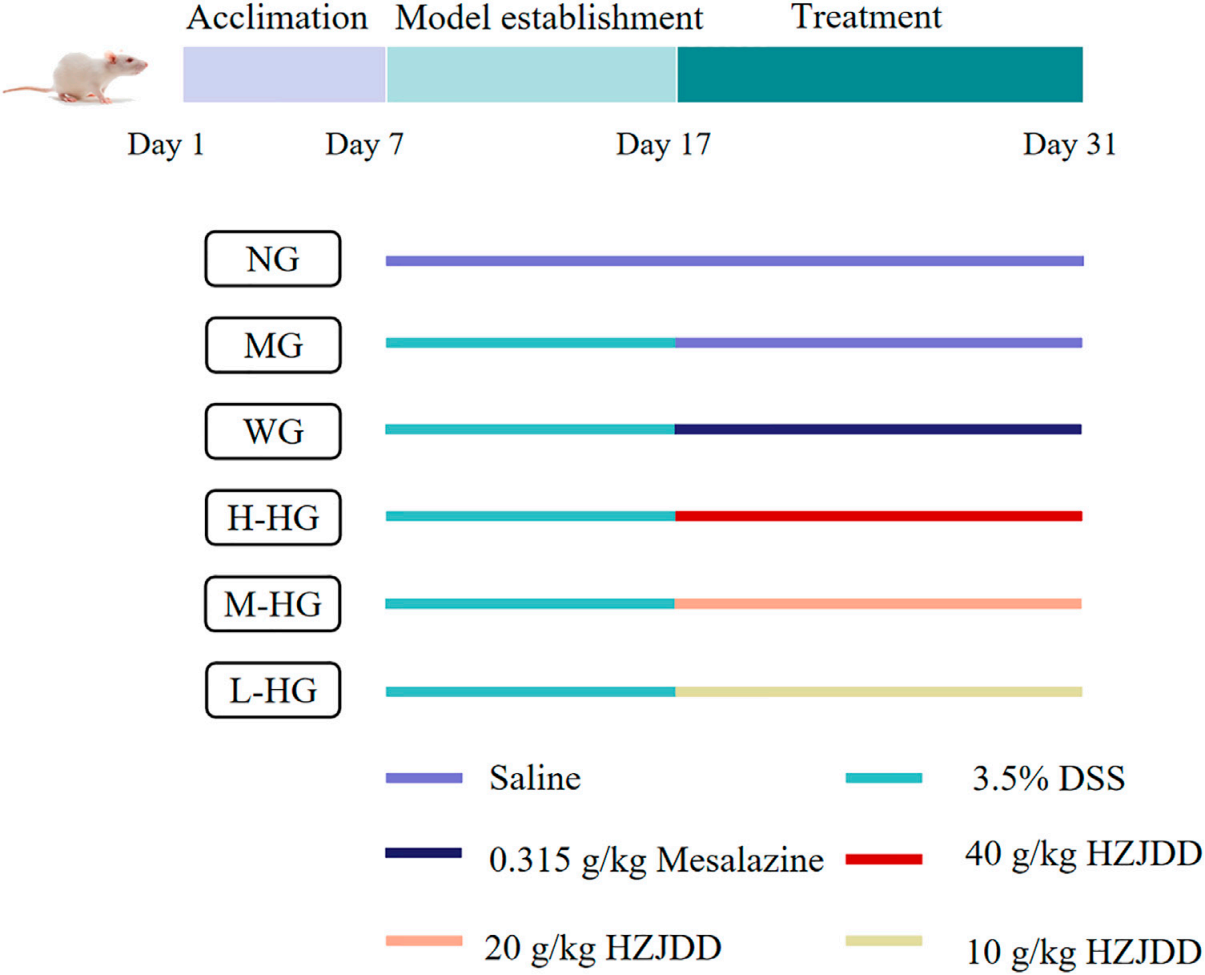
The experimental design diagram.

**FIGURE 4:**
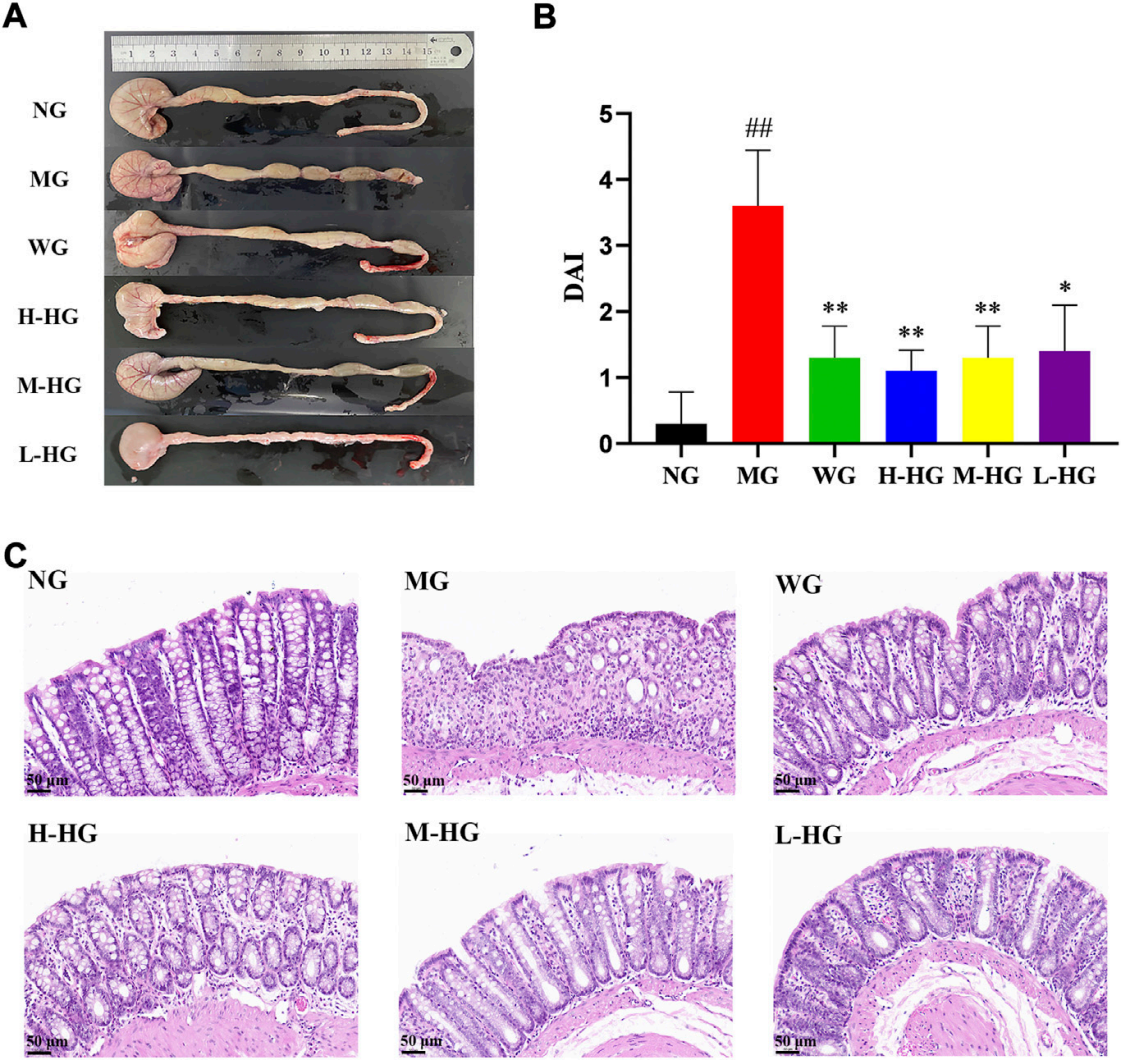
HZJDD alleviated DSS-induced UC in rats. **(A)** The length of the colon in each group. **(B)** Disease activity index (DAI) in each group (n = 10). **(C)** Results of HE staining (×200) in colon tissues of each group. ^##^ *p* < 0.01 vs NG, ^**^ *p* < 0.01 or ^*^ *p* < 0.05 vs MG.

After modeling and administration (Figure 3), as shown in Figure 4A, H-HG and M-HG could significantly alleviate colon shortening. Compared with NG, the DAI score of MG was significantly increased. Compared with MG, the DAI scores of HZJDD treatment groups decreased significantly, suggesting that HZJDD could reverse the increasing trend of DSS-induced UC in rats (Figure 4B). Histopathological results showed that the colon tissue structure of NG was normal without obvious pathological changes; the glands of MG were disorderly arranged, with shallow ulcers formed, and inflammatory cells were diffusely distributed. After HZJDD treatment, colon injury was improved in all groups, with a regular arrangement of glands and mucosal inflammatory cell infiltration area decreased in H-HG and M-HG (Figure 4C).

#### 3.2.2 HZJDD improved the microstructure of DSS-induced UC in rats

**FIGURE 5:**
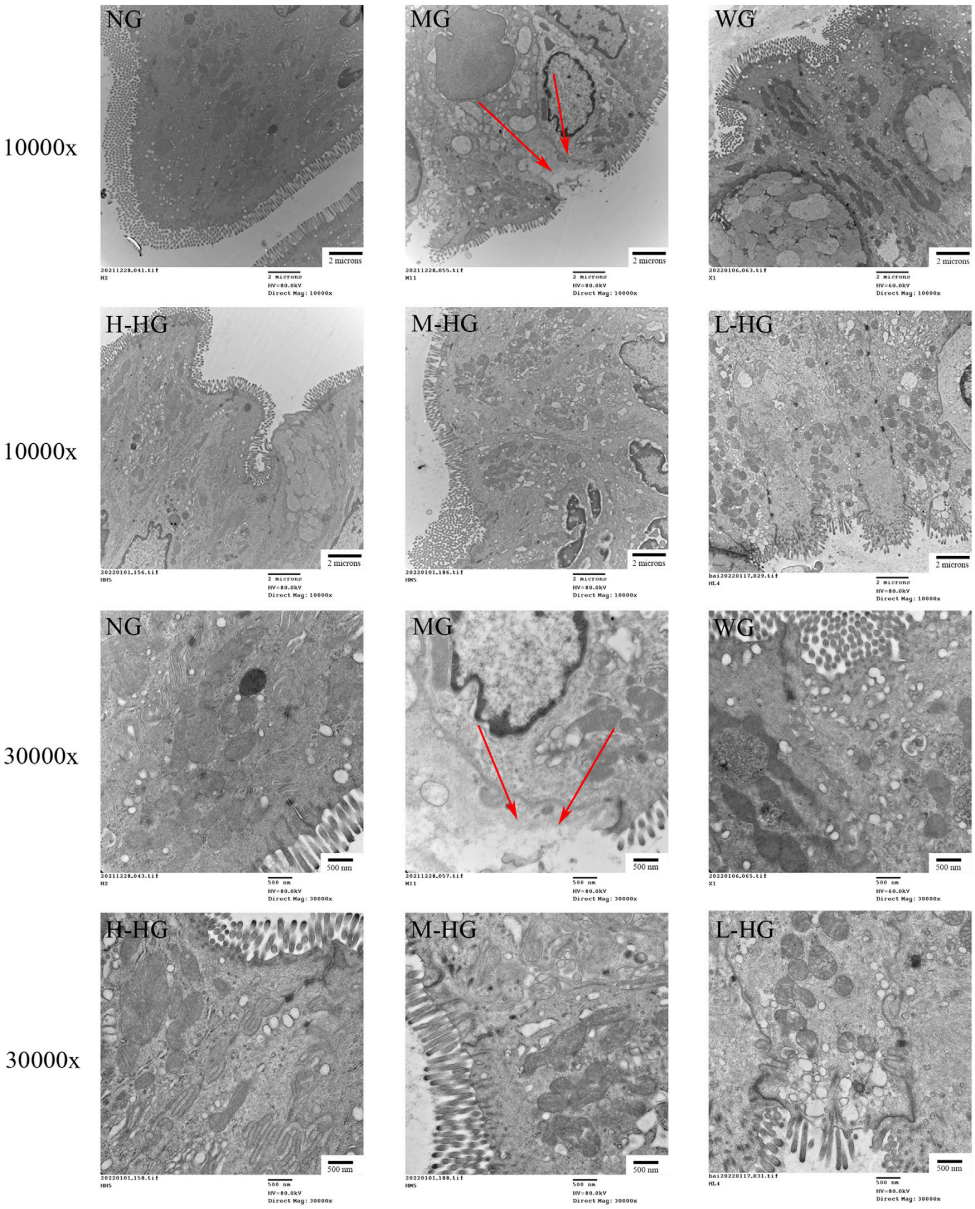
HZJDD ameliorated ultrastructural changes in DSS-induced UC in rats (n = 3). The arrow indicated that cell membrane lysis and ruptured with cytoplasmic spillage. Scale bar = 2 μm (10,000x) or 500 nm (30,000x).

The NG cells were arranged neatly, cells were tightly connected, and mitochondria had abundant normal cristae. MG showed cell membrane lysis and ruptured with cytoplasmic spillage, mitochondrial swelling, cristae fragmentation, and residual binding vacuoles. Compared with MG, the cells in each treatment group were neatly arranged, the cell connections were tighter, and the mitochondrial structures were repaired (Figure 5).

#### 3.2.3 HZJDD inhibited the inflammatory response of DSS-induced UC in rats

**FIGURE 6:**
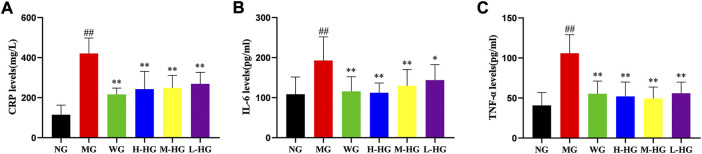
HZJDD inhibited the inflammatory response of DSS-induced UC in rats. The levels of CRP **(A)**, IL-6 **(B)** and TNF-α **(C)** in each group. Data are presented as the mean ± SD (n = 10). ^##^ *p* < 0.01 vs NG, ^**^ *p* < 0.01 or ^*^ *p* < 0.05 vs MG.

As shown in Figure 6, compared with NG, the levels of CPR, IL-6, and TNF-α in MG rats were significantly increased. Compared with MG, the levels of CPR, IL-6, and TNF-α in HZJDD treatment groups were markedly down-regulated.

#### 3.2.4 HZJDD modulated oxidative stress of DSS-induced UC in rats

**FIGURE 7:**
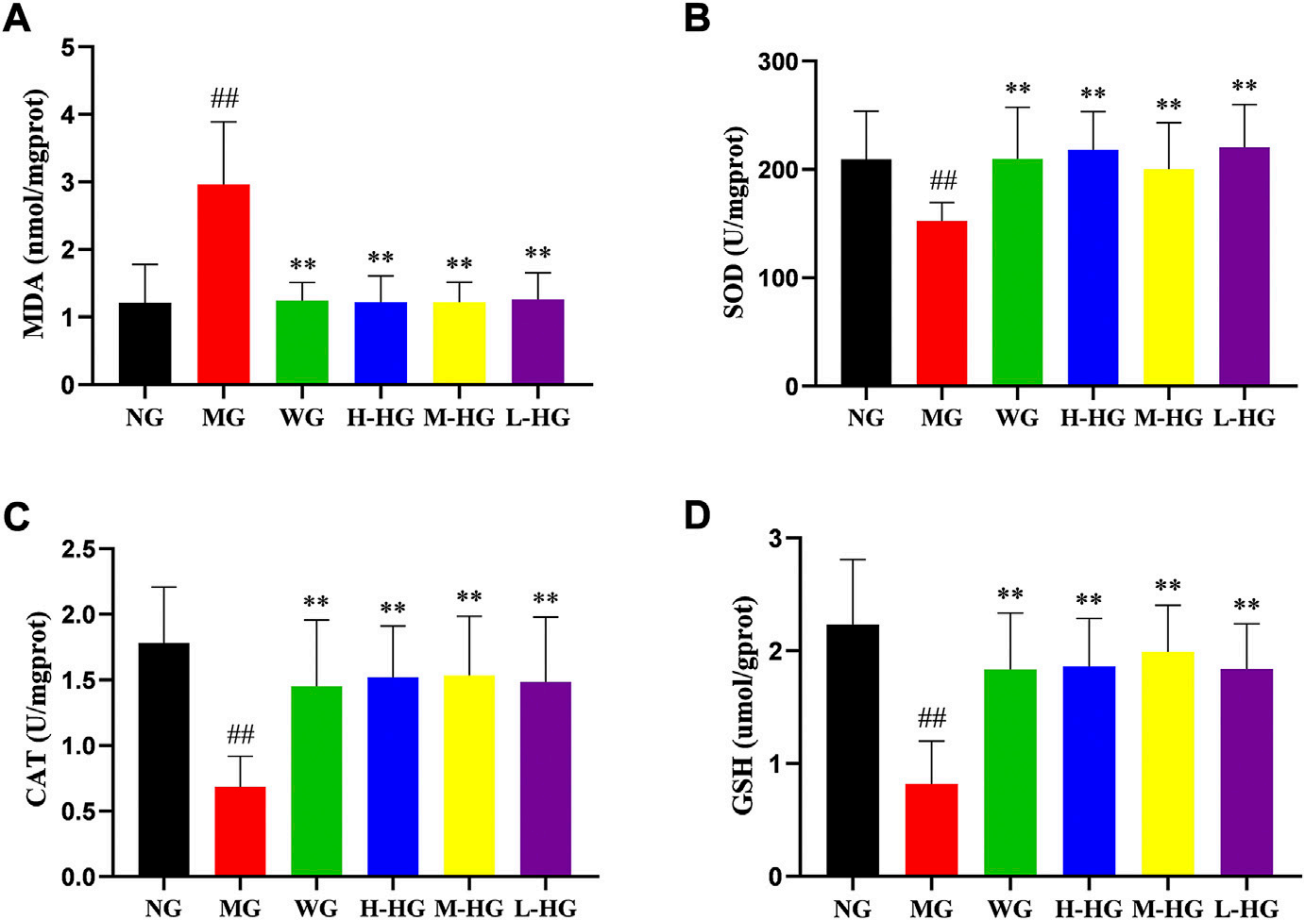
HZJDD modulated oxidative stress of DSS-induced UC in rats. The activities of MDA **(A)**, SOD **(B)**, CAT **(C)** and GSH **(D)** in each group. Data are presented as the mean ± SD (n = 10). ^##^ *p* < 0.01 vs NG, ^**^ *p* < 0.01 or ^*^ *p* < 0.05 vs MG.

Figure 7 showed that compared with NG, DSS increased the level of oxidative stress in MG by decreasing CAT, GSH, and SOD levels and increasing MDA levels. Compared with MG, HZJDD showed stronger antioxidant capacity by increasing CAT, GSH, and SOD levels and decreasing MDA levels. It is noteworthy that all HZJDD groups showed strong antioxidant capacity.

#### 3.2.5 HZJDD restrained the NLRP3/caspase-1 pathway of DSS-induced UC in rats

**FIGURE 8:**
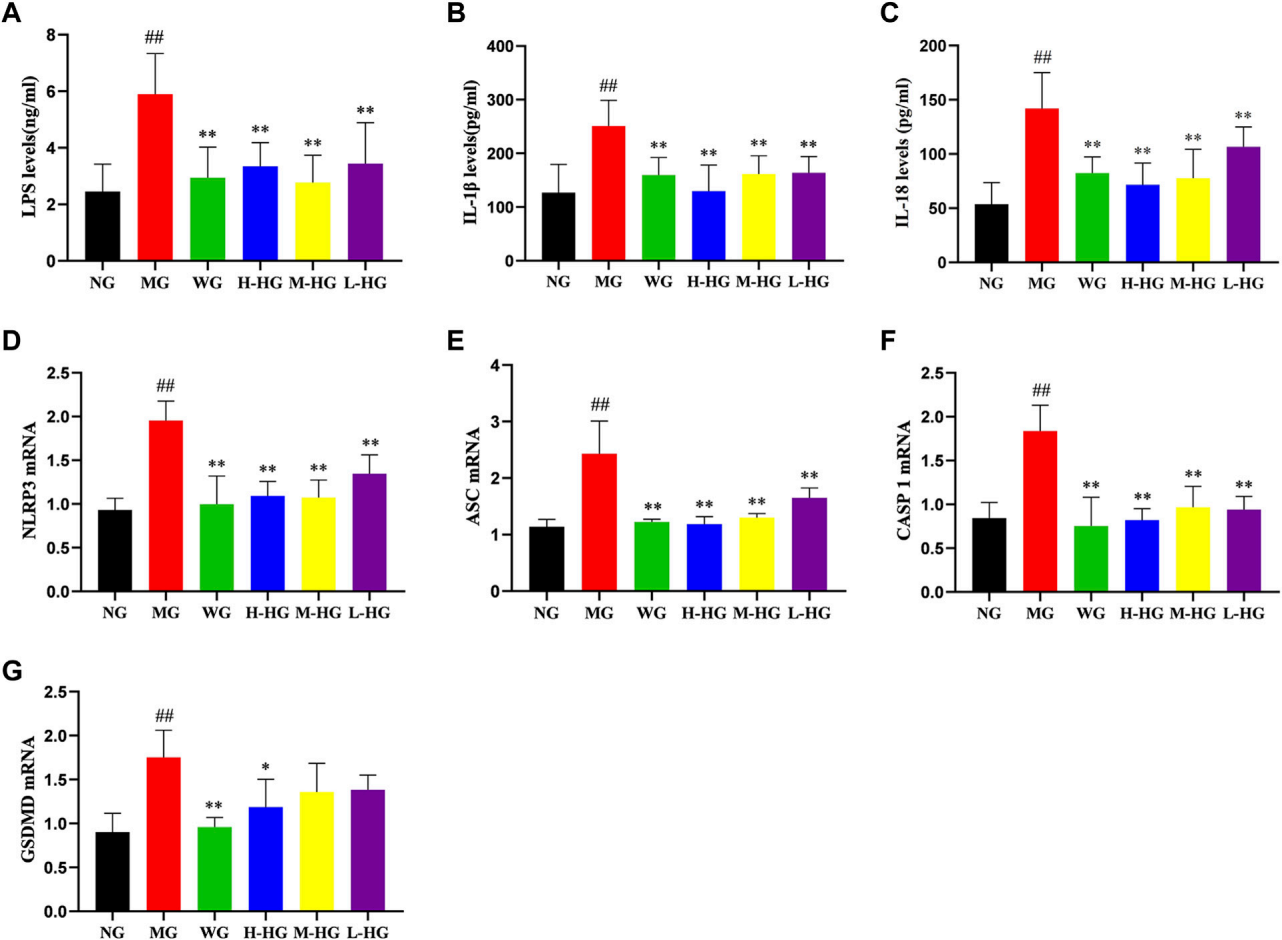
HZJDD restrained the NLRP3/caspase-1 pathway of DSS-induced UC in rats. The levels of LPS **(A)**, IL-1β **(B)**, and IL-18 **(C)** (n = 10) and the mRNA expression of NLRP3 **(D)**, ASC **(E)**, caspase-1 **(F)**, and GSDMD **(G)** (n = 3) in each group. Data are presented as the mean ± SD. ^##^ *p* < 0.01 or ^#^ *p* < 0.05 vs NG, ^**^ *p* < 0.01 or ^*^ *p* < 0.05 vs MG.

**FIGURE 9:**
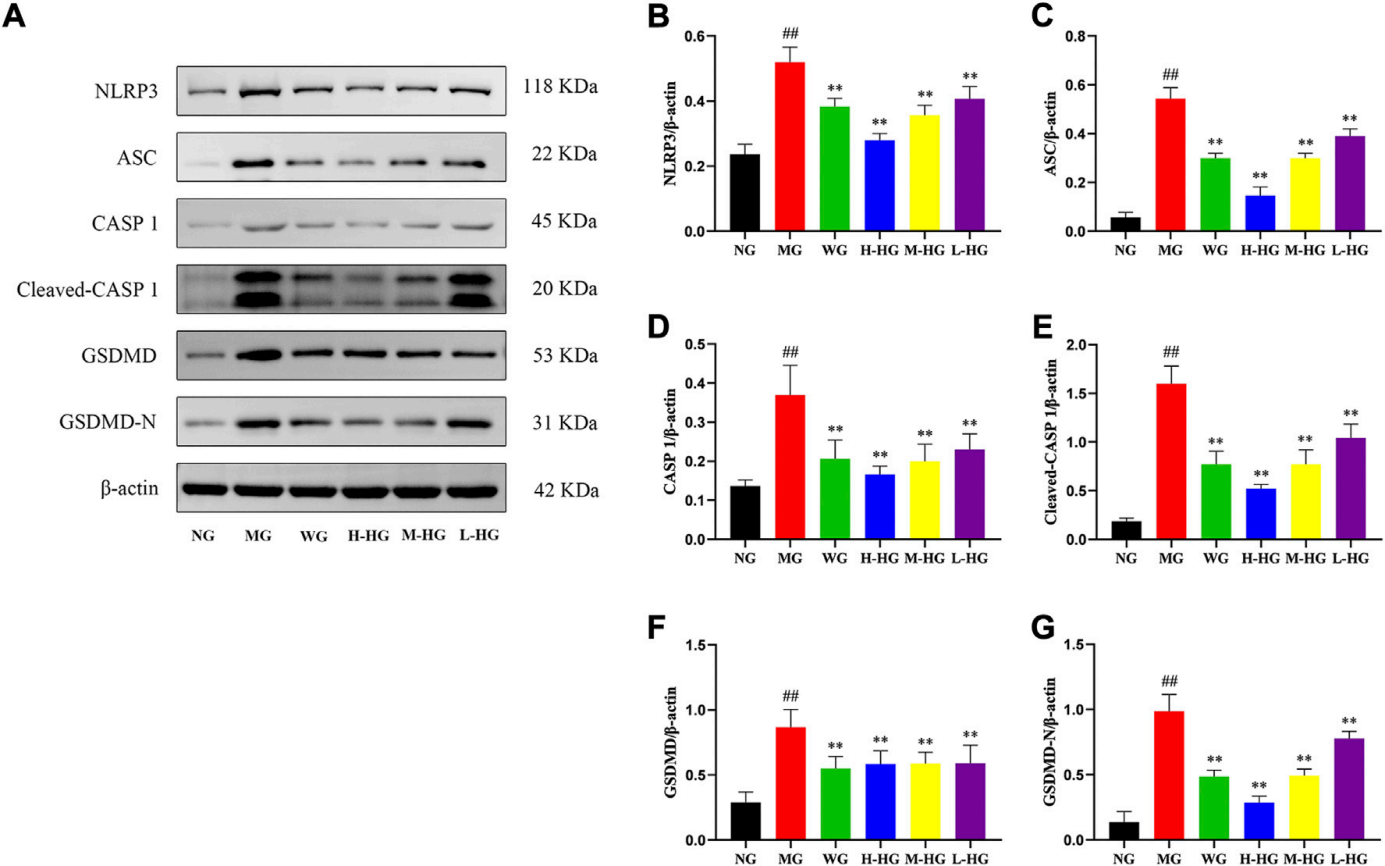
HZJDD restrained the NLRP3/caspase-1 pathway of DSS-induced UC in rats at the protein level **(A)**. Relative protein levels of NLRP3 **(B)**, ASC **(C)**, caspase-1 **(D)**, cleaved-caspase-1 **(E)**, GSDMD **(F)** and GSDMD-N **(G)** were normalized to β-actin. Data are presented as the mean ± SD (n = 3). ^##^ *p*＜0.01 vs NG, ^**^ *p* < 0.01 or ^*^ *p* < 0.05 vs MG.

To further clarify the effect of HZJDD, we analyzed the expression level of LPS related NLRP3/caspase-1 signaling pathway. ELISA results indicated that compared with MG, the expression levels of LPS, IL-1β, and IL-18 in HZJDD groups were significantly decreased (Figures 8A–C). Western blotting results indicated that compared with MG, the levels of NLRP3, ASC, caspase-1, cleaved-caspase-1, GSDMD, and GSDMD-N were significantly decreased in HZJDD groups (Figure 9). We also examined the expression of these genes in colon tissue by qRT-PCR. The results indicated that HZJDD reduced the expression of these genes (Figures 8D–G). These results suggested that HZJDD attenuates DSS-induced UC by inhibiting the NLRP3/caspase-1 pathway.

**FIGURE 10:**
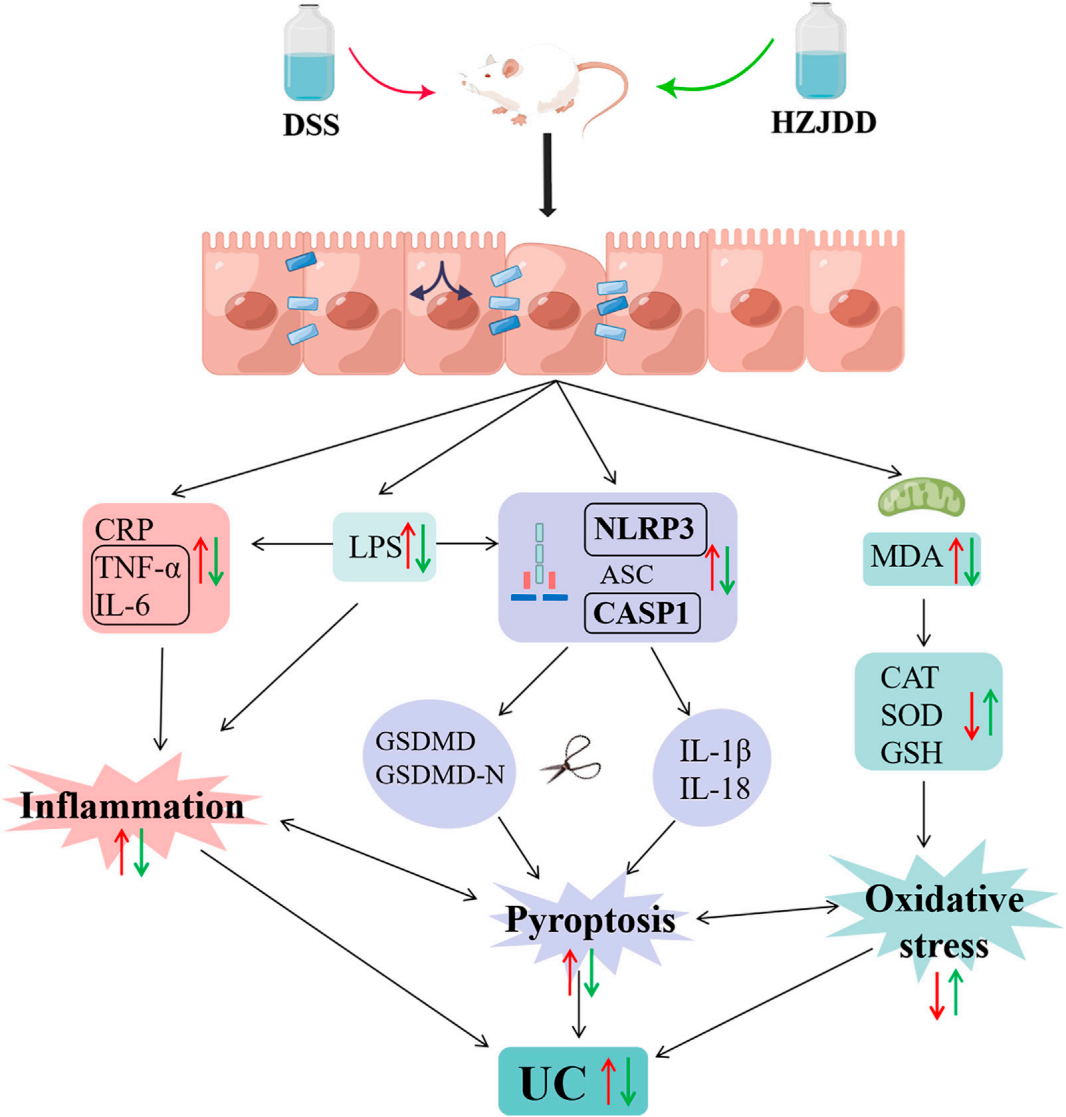
The protective mechanism of HZJDD against DSS-induced UC (this image was created using Figdraw).

## 4 Discussion

Because the pathogenesis of UC is still unclear, and the disease is chronic and prone to recurrence, it is difficult to treat (Ishida et al., 2021). The side effects of 5-ASA and immunosuppressants commonly used in clinics affect the patient’s mental state and normal work and life (de Mattos et al., 2015; Jie et al., 2021). Naturally derived Chinese medicinal materials have fewer side effects, more components, and multiple targets and unique advantages in treating UC (Montrose et al., 2011). In TCM theory, HZJDD has the advantages of clearing heat and detoxifying and can significantly relieve many symptoms of UC, including irregular stools, blood in the stool, and fever. It has potential in the treatment of gastrointestinal diseases, such as ulcerative colitis. The healing effects of this traditional herb are believed to come from its complex biochemical components. It is worth mentioning that some active components in HZJDD have been shown to have anti-inflammatory and anti-oxidant effects (Yuan et al., 2019; Miao et al., 2020; Rao et al., 2022). In this study, we comprehensively analyzed the potential mechanism of HZJDD in treating UC by integrating network pharmacology and animal experiments and provided evidence for further application.

The network pharmacology results suggested that quercetin, kaempferol, beta-sitosterol, stigmasterol, luteolin, and isorhamnetin may be the main active components of HZJDD for the treatment of UC, which is consistent with previous studies. Quercetin (Carrasco-Pozo et al., 2016; Wang et al., 2021c), kaempferol (Rajendran et al., 2014; Qu et al., 2021), beta-sitosterol (Han et al., 2014; Shou et al., 2022), stigmasterol (Han et al., 2022; Jia et al., 2022), luteolin (Seelinger et al., 2008; Franza et al., 2021), and isorhamnetin (Yang et al., 2013; Dou et al., 2014) have strong anti-inflammatory, anti-oxidant, and gastroprotective effects. They reduce DSS-induced colitis by inhibiting various inflammatory factors.

By further analysis, we found that six of the first 15 biological processes belonged to inflammatory response, cellular response to hypoxia, and cellular response to LPS. It is suggested that the anti-UC mechanism of HZJDD may be related to inflammation, oxidative stress, and the regulation of LPS. We constructed a network diagram of GO-BP biological processes and intersection targets and found that TNF-α and IL-6 were related to inflammatory response; SOD and CAT were related to oxidative stress; and IL-6 and IL-1β were related to LPS-induced cellular responses. In recent years, inflammation, oxidative stress, and LPS-induced cellular responses have been recognized as key factors affecting the formation and progression of UC (Bai et al., 2019; Qu et al., 2021).

LPS is an endotoxin derived from Gram-negative bacteria. When LPS accumulates in the intestinal mucosa, it will activate macrophages and cause the leakage of neutrophils, resulting in damage to the intestinal mucosal epithelium (Li et al., 2018). The infiltration of large numbers of neutrophils into the intestinal mucosa leads to the accumulation of pro-inflammatory factors, such as TNF-α, IL-6, IL-1β, and oxidative stress (Baradaran et al., 2019). Notably, LPS-induced inflammatory processes have been demonstrated to cause activation of the NLRP3 inflammasome pathway to induce pyroptosis (Lv et al., 2017). The current study confirmed that DSS mice could lead to the activation of the NLRP3/caspase-1 pathway to damage the intestinal mucosa and induce the occurrence of UC (Sun et al., 2021). In addition, there is evidence that inflammation and oxidative stress may be important factors triggering pyroptosis (Hou et al., 2021). Inducing cell death and releasing inflammatory factors, pyroptosis amplifies inflammation and oxidative stress (Qiu et al., 2019), aggravates intestinal mucosal damage, and forms a vicious circle. Inflammation, oxidative stress, and pyroptosis are inseparable from the formation of UC. Consequently, to explore the mechanism of HZJDD in treating UC, we examined the biological processes of inflammation, oxidative stress, and LPS related NLRP3/caspase-1 pyroptosis signaling pathway (Figure 10).

DSS-induced UC rats were used in this study. DSS is a chemical colitogen with anticoagulant properties, which has been widely used in UC modeling due to its rapidity, reproducibility, and controllability (Chassaing et al., 2014). It is well known that the DAI score is an evaluation metric for assessing DSS-induced UC models (Oliveira et al., 2014; Amiriani et al., 2018). Our results indicated that DAI scores were significantly elevated in MG compared with NG. Histopathology showed that the glandular arrangement was disordered with the formation of shallow ulcers in DSS-induced in UC rats. In addition, electron microscope results suggested that the cell junction gap was significantly wider in MG than in NG, the cell membrane was dissolved and ruptured, cytoplasm overflowed, mitochondria were swollen, and cristae were broken. Based on the above results, we had successfully established the DSS-induced UC rats. In contrast, HZJDD treatment reversed DSS-induced UC in rats, as demonstrated by improved colon length, reduced DAI score, and histomorphology and microscopic morphology restoration.

The inflammatory response cannot be ignored in the pathogenesis of UC, so strict control of the inflammatory response is the key strategy for treatment. CRP is a non-specific marker for monitoring the severity of UC disease (Sayar et al., 2020) and can positively respond to inflammatory factors, including IL-6 and TNF-α (Walsh et al., 2016). Studies have found that the levels of TNF-α in the blood, mucous membranes, and feces of UC patients are increased (Murch et al., 1991; Braegger et al., 1992). Some scholars believe that IL-6 is the central link to UC, and the serum level of IL-6 can predict the risk of relapse after hormone-induced remission (Ali et al., 2021). In this study, we observed that HZJDD remarkably reduced the level of IL-6, CRP, and TNF-α in DSS-induced colitis rats, indicating that HZJDD could exert anti-inflammatory effects against DSS-induced in UC rats.

Numerous studies have shown that the interaction of inflammation and oxidative stress may be a significant part of inducing UC (Wang et al., 2019; Jeon et al., 2020). Inflammation augments oxidative stress by stimulating reactive oxygen/nitrogen species-generating systems, and oxidative stress is intimately involved in the execution of inflammatory cytokines and infiltration of inflammatory cells (Rezaie et al., 2007; Zhu and Li, 2012). LPS induces mitochondrial production of free radicals (Zhang et al., 2021), and the released free radicals cause an imbalance between oxidants and antioxidants, activate inflammatory mediators, and disrupt the intestinal mucosal barrier (Zhou et al., 2006). Superoxide and peroxide are sources of reactive oxygen species in inflammatory mucosa (Elmaksoud et al., 2021), and CAT, GSH, and SOD reduce oxidative stress response by decomposing superoxide and peroxide (Hagar et al., 2007; Li B. et al., 2021). The content of MDA (the final product of lipid peroxidation) is proportional to lipid peroxidation, which can indirectly manifest the content of oxygen free radicals in the body and the degree of cell damage (Tian et al., 2017). Therefore, we measured the contents of CAT, GSH, SOD, and MDA in colon tissues to evaluate the antioxidant activity of HZJDD. Interestingly, in the present study, we observed oxidative stress in DSS-induced UC rats, which was confirmed by increased contents of MDA and decreased contents of CAT, GSH, and SOD. However, post-treatment with HZJDD showed that the content of MDA was decreased, and the contents of CAT, GSH, and SOD were increased.

Pyroptosis is an innovative inflammatory programmed cell death accompanied by the release of IL-1β and IL-18 (Pellegrini et al., 2020; Liu F. et al., 2021), which are the main pro-inflammatory factors leading to UC (Nowarski et al., 2015; Chen and Simmons, 2019). Pyroptosis is associated with the activation of NLRP3, which binds to ASC to recruit caspase-1 to form an inflammatory complex. Activated caspase-1 cleaves GSDMD to expose its N-terminal active domain, and GSDMD-N translocates to the plasma membrane to punch holes, resulting in swelling and rupture of the cell membrane, accompanied by the release of IL-1β and IL-18 (Wu et al., 2020). In addition, some scholars discovered that inhibition of NLRP3 inflammasome-dependent pyroptosis could effectively improve DSS-induced UC (Liu C. S. et al., 2021). The characteristics of pyroptosis are cell swelling, cell membrane rupture, and release of pro-inflammatory factors (Yuan et al., 2018), which were proved by the electron microscopy results in this experiment.

Furthermore, the expressions of LPS, IL-1β, and IL-18 were elevated in DSS-induced UC in rats. To further determine whether the pyroptosis process was activated after the increase of LPS, we examined the expression of the NLRP3/caspase-1 signaling pathway at the protein and gene levels. The results demonstrated that DSS-induced UC rats showed activation of pyroptosis, which could be derived from the upregulation of protein and gene expressions of NLRP3, ASC, caspase-1, and GSDMD. On the other hand, HZJDD intervention suppressed the overexpression of LPS and downregulated the expression of NLRP3, ASC, caspase-1, and GSDMD at the protein and gene levels. These results indicated that HZJDD could inhibit the NLRP3/caspase-1 signaling pathway to suppress the inflammatory response and pyroptosis.

The study examined the exact role and potential mechanisms of HZJDD for the treatment of UC. Meanwhile, Pharmacokinetic study, including absorption, distribution, metabolism and excretion processes, is indispensable to establish concentration-activity relationship and facilitate target identification of HZJDD. However, HZJDD is a multi-component, multi-target complex prescription drug, therefore, pharmacokinetic studies of HZJDD will be the focus of our future studies.

## 5 Conclusion

In conclusion, our study employed network pharmacology and experimental validation methods to identify the therapeutic targets and potential mechanisms of HZJDD. Network pharmacology results suggested that the multi-target synergistic mechanism of HZJDD in the treatment of UC may be related to inflammation, oxidative stress, and the regulation of LPS. Animal experiments showed that HZJDD exerted a therapeutic effect on DSS-induced UC rats by reducing inflammation, oxidative stress, and restraining the NLRP3/caspase-1 signaling pathway to inhibit pyroptosis. Our study provides new ideas for treating UC with HZJDD and offers hope for herbal-based complementary and alternative treatments for UC.

## Data Availability

The raw data supporting the conclusions of this article will be made available by the authors, without undue reservation.
