# Supplementary material for: The potential mechanism of huazhuojiedu decoction in the treatment of ulcerative colitis based on network pharmacology and experimental validation
Source: Front Pharmacol. 2022 Oct 14;13:1033874. doi: 10.3389/fphar.2022.1033874 (PMC9614068; doi:10.3389/fphar.2022.1033874)
Supplement: Supplementary file 1 [file DataSheet1.zip › Supplementary Table S1.DOC]

| Score | body weight loss | diarrhea | hematochezia |
| --- | --- | --- | --- |
| 0 | none | normal | no bleeding |
| 1 | 1 %-5 %loss | — | — |
| 2 | 5 %-10 %loss | loose stools | slight bleeding |
| 3 | 10 %-20 % loss | — | — |
| 4 | over 20 % loss | watery diarrhea | gross bleeding |

Supplementary Table S1 The standard of DAI score.
