# Supplementary material for: The potential mechanism of huazhuojiedu decoction in the treatment of ulcerative colitis based on network pharmacology and experimental validation
Source: Front Pharmacol. 2022 Oct 14;13:1033874. doi: 10.3389/fphar.2022.1033874 (PMC9614068; doi:10.3389/fphar.2022.1033874)
Supplement: Supplementary file 1 [file DataSheet1.zip › Supplementary Table S2.DOC]

Supplementary Table S2 Primer sequence of QRT-PCR.

| Gene |  | Primer Sequences (5′–3′) | Length (bp) |
| --- | --- | --- | --- |
| NLRP3 | sense | GATTTCTCCACAACTCACCCAA | 112 |
|  | antisense | AGTCTGGAAGAACAGGCAACAT |  |
| ASC | sense | CTGTGCTTAGAGACATGGGCA | 95 |
|  | antisense | AGGGACACTGGTTGCAGTAG |  |
| Caspase-1 | sense | TGCCTGGTCTTGTGACTTGGAG | 132 |
|  | antisense | TGTCCTGGGAAGAGGTAGAAACG |  |
| GSDMD | sense | CAGGCAGCATCCTTGAGTGTC | 186 |
|  | antisense | CCAAGACGTGCTTCACCAACT |  |
| GAPDH | sense | CTGGAGAAACCTGCCAAGTATG | 138 |
|  | antisense | GGTGGAAGAATGGGAGTTGCT |  |
